# Supplementary material for: The impact of human dispersals and local interactions on the genetic diversity of coastal Papua New Guinea over the past 2,500 years
Source: Nat Ecol Evol. 2025 Jun 4;9(6):908–23. doi: 10.1038/s41559-025-02710-x (PMC12148941; doi:10.1038/s41559-025-02710-x)
Supplement: Supplementary file 1 — Modelling of the split time between Eriama and Nebira and Supplementary Tables 1–14 index. [file 41559_2025_2710_MOESM1_ESM.pdf]

nature ecology & evolution

Supplementary information https://doi.org/10.1038/s41559-025-02710-x

The impact of human dispersals and local interactions on the genetic diversity of coastal Papua New Guinea over the past 2,500 years

In the format provided by the authors and unedited

Index

Ethical considerations for the analysis of the ancient genomes 2
Terminology 3
Site descriptions
Reber-Rakival, Watom (SAD, SAC) 6
Tilu (JCA) 8
Nunguri 9
Nebira 2 (ACJ) 10
Eriama (ACV) 11
Material and Methods
Modeling of the split time between Eriama and Nebira 12
AMS and Isotope Analysis 25
References 15
Supplementary Data Tables Index 18

Ethical considerations for the analysis of the ancient genomes.
Today, Papua New Guinea's communities are highly structured by a huge linguistic and cultural diversity. The patterns observed today likely have roots in the distant past, but are also influenced by dynamics rooted in colonial interactions and imperialistic efforts in the present day. The combination of a high abundance of natural resources and the increased interest of WEIRD (Western, Educated, Industrialized, Rich, Democratic) countries has led to political tensions between various groups, lamentably often culminating in violence (Forsyth, Kipong et al. 2024). In such a political climate, one must consider the potential impacts of certain results when linking the past to the present.

This study uses ancient genomes to investigate the genetic diversity in the past, and discusses underlying dynamics by integrating other types of data such as isotopes and AMS-dating, and contextualizing with archaeological and linguistic evidence. The majority of individual genomes produced for this study is of high quality, allowing imputation of missing sites and fine scale analyses within and between groups with the potential to link the ancient individuals to present-day individuals who have donated their genomes to science.

For this study, we have purposefully chosen to not attempt to directly link the ancient individuals to their potential descendants in the region today. While the data in this study adds significantly to our understanding of the past genetic diversity, only two sites with n=22 (Nebira) and n=8 (Eriama) individuals qualify for an analysis of direct links from the past to the present. The low number of individuals, and the small geographic area they cover will certainly not be representative of the past genetic diversity for the entire South Coast where the population history before and after colonial invasion is much more complex. Additionally, the linguistic data recorded for the present-day comparative datasets suggests that among the sampled present-day individuals, only Motu speakers donated samples. Additionally, the ethnographic records rely on the statements of the participants, which could be unprecise or even wrong. Linking the few ancient individuals directly to present day groups, i.e. through IBD analysis (see p.24 and following), could potentially lead to historical misinterpretations about land in a present-day context.

These factors were considered when discussing whether data should be made publicly available, as is the case in almost 100% of ancient DNA studies (Anagnostou, Capocasa et al. 2015), and expected from other researchers in the field (Alpaslan-Roodenberg, Anthony et al. 2021, Bergström 2023). We have decided to make the data publicly available, but purposefully not conducted an analysis potentially able to directly link the ancient and modern individuals from the region. These comparisons could be undertaken in future with a community-driven research project.

We strongly recommend other researchers interested in such analyses to carefully consider the control they have over the dissemination and

communication of the results, how they can be interpreted or mis-interpreted, and how they will affect the lives of people part of the communities on the South Coast of Papua New Guinea.

Terminology

The Pacific region has a long and complex cultural and biological history. It was shaped through multiple dispersal events, followed by genetic admixture and cultural evolution, but also greatly impacted by European colonization starting with Vasco Núñez de Balboas voyages to the Pacific in 1530. As observed in many places of the world, European invasion had a lasting impact on the cultural and biological landscape of the Pacific, imposing their foreign culture, religion and languages on the people of the region, renaming and naming people and places from Eurocentric and racist viewpoints. This led to a subdivision that only at first glance seems reasonable. The region was divided into Micronesia ("the small islands") Polynesia ("the many islands") and Melanesia ("the black islands"). While Poly- and Micronesia are based on a topographical description of the islands, Melanesia, comprising New Guinea, the Bismarck Archipelago and the island groups of Fiji, New Caledonia, Vanuatu and Solomon Islands, referred to the color of people's skin. Additionally, the concepts of Micronesia and Melanesia have proven inadequate especially in archaeological contexts (Terrell 1986, Thomas, Abramson et al. 1989, Green 1991), as the divisions are not rooted in the pre-contact history (Green 1991). People inhabiting this region today partly have reclaimed the word (Kabutaulaka 2015) and are challenging negative representation perpetuated since colonial times. However, we are unable to infer how people in ancient times self-identified and have to assume the term introduced by the colonizers homogenised the different peoples based on the color of their skin, ignoring diverse cultures and identities.

Taking into consideration the biogeography, linguistics and ethnography of the region there is no useful application of the term Melanesia (Green 1991), hence a perpetuation of this racially inspired term can be avoided. Genetically some populations included in the Melanesian geographical sphere derive their main ancestry from a population that settled in the region ~45,000 years ago. This ancestry signal is maximised in populations inhabiting the highlands of Papua New Guinea. However, a substructure can be observed spreading along a cline without clear grouping of the different islands or archipelagos. We therefore chose to refer to a genetic component that is linked to people from New Guinea, the Bismarck Archipelago or the Solomon Islands as "Papuan-related" if a more general term is needed. We use the names of islands or archipelagos in which a certain genetic signal is maximized in a more regional analysis, and if the signal is specific to a certain group on said islands, e.g. for Baining from New Britain we refer to the genetic ancestry as specifically related to those groups, e.g. "Baining-related".

The people associated with the Lapita Cultural Complex were the first to settle on New Caledonia, Vanuatu, Tonga, Fiji and Samoa. Their genetic ancestry has been shown to derive from an East-Asian ancestral population most similar to ancient and present-day Indigenous individuals from Taiwan and the Philippines (Skoglund, Posth et al. 2016), but show a distinct genetic profile. Although the distinctness of material culture and its great similarity across the islands implies also a cultural unity, it is impossible to reconstruct how people in the past identified, and the vast distance between Vanuatu and Tonga might even suggest different identities despite the same origins and material culture. However, seeing the genetic similarity of the first settlers of both Vanuatu and Tonga, we are in need of a term to refer to groups in a more general way. Oftentimes, studies revert to using the term for the archaeological culture associated with a certain genetic signal. Referring to the genetic composition of the first inhabitants of Vanuatu and Tonga as “Lapita” assumes a connection of material culture, identity and genetic composition we do not want to imply. Other genetic studies have attempted to resolve this by use of the term “First Remote Oceanians” (Skoglund, Posth et al. 2016, Lipson, Skoglund et al. 2018).

However, Remote Oceania not only covers the islands east of the Solomon Islands, but also includes also the islands of Micronesia, north of New Guinea. Based on the similarities of pottery styles and decorations, a dispersal “from the Philippines via the Mariana Islands” to the Bismarck Archipelago has been proposed before (Bellwood 2007). Radiocarbon dates of ceramic artefacts found in the Mariana Islands date the initial settlement of humans to 3500 - 3200 BP (Hung, Carson et al. 2011, Carson and Kurashina 2012, Petchey, Clark et al. 2018, Carson 2020, Petchey and Clark 2021), according to palaeo-environmental evidence even earlier (Athens and Ward 2004), suggesting the settlement occurred at the same time or even earlier to that of the Bismarck Archipelago. Recent genetic studies investigated the genetic make-up of ancient inhabitants of Guam and Saipan, dating from 2200 BP (Pugach, Hubner et al. 2021, Liu, Hunter-Anderson et al. 2022). Ancestry modeling of these individuals together with other populations in the Pacific have shown that their ancestry derives from a lineage ancestral to that of the individuals from Vanuatu and Tonga. Based on the archaeological and genetic evidence available, it is likely that the Mariana Islands were settled before Vanuatu and Tonga, possibly making the ancestors of the individuals from Guam and Saipan the “*First* Remote Oceanians”. As future studies will likely include more ancient individuals not only from the southern and eastern parts of Remote Oceania, but also northern Remote Oceania, terminology describing genetic signals has to be reconsidered. To not confuse the geographical anchors involved in the settlement of Remote Oceania, we refer to the genetic profile as observed in the skeletons dating to the Lapita period in Vanuatu and Tonga, as “Early Remote Oceanian-related”.

A similar problem arises when describing the general genetic profile deriving from a dispersal from Asia. In this study, we refer to the genetic component associated with a dispersal from the mainland and island South East Asia as “East Asian-related”,

similar to the oversimplification of the diverse Papuan ancestry profiles to “Papuan-related. This term should not be understood as a reflection of a sociopolitical region, but inspired by the broad geographical region that is most likely the ancestral home of the genetic profile dispersing in East-, South East- and Island South East Asia ~ 7000 -500 years ago. Alternative terms could be “Indigenous East Asian”. However, since mostly Taiwanese and Philippine indigenous genomes were used for or the modeling this would disregard the many indigenous East Asian groups with different genetic profiles. Considering the use of “Austronesian” for the genetic signal, we also decided to avoid this language-based naming because Austronesian speaking groups today are very variable in their genetic ancestry, the cultural representation and geographic extent of the language.

Site descriptions

Reber-Rakival (SAD and SAC)

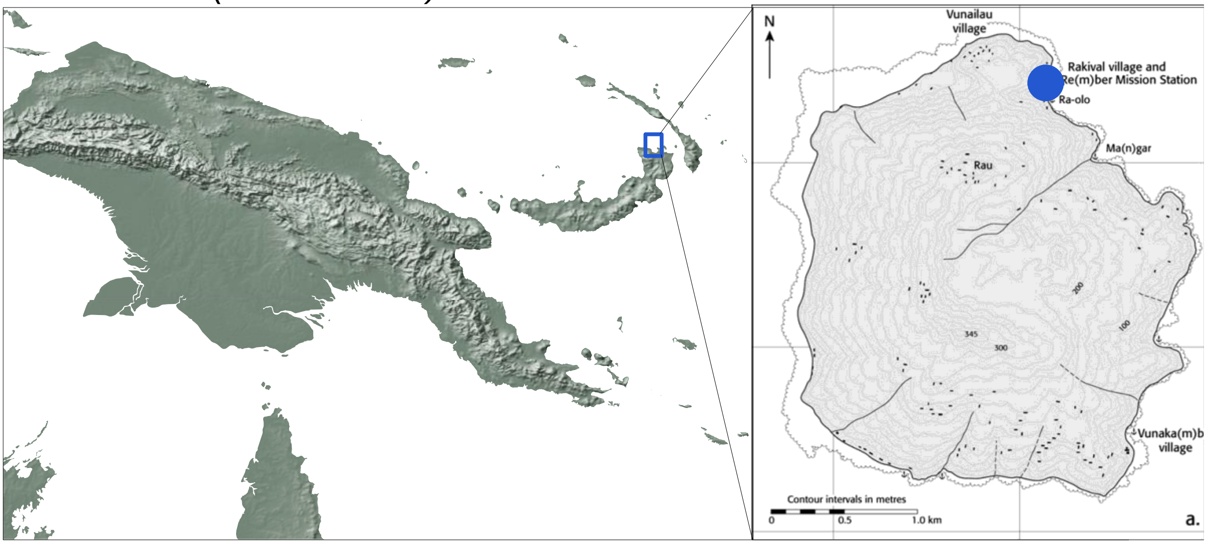

Supplementary Figure 1: Map of PNG and Watom Island showing location of sites.

The Reber-Rakival site is located on Watom Island, north of East New Britain, 9 km off the Gazelle Peninsula in the Bismarck Sea. A number of sites containing Lapita pottery were excavated on Watom between 1965 and 2009, including the SAC and SAD sites from Reber-Rakival detailed in this study (Allen 1984, Green, Anson et al. 1989, Anson 2000, Green and Anson 2000, Anson, Green et al. 2005). Out of a total of 14 individuals recovered, five are analyzed here. Multiple radiocarbon dates, previously produced from human bone from the Watom burials (ca. 2800–2350 cal BP) (Petchey and Green 2005, Athfield, Green et al. 2008, Petchey, Spriggs et al. 2011), placed these individuals in the Middle/Late Lapita phases in the Bismarck Archipelago (ca. 3000–2800/2700–2200 BP) (Summerhayes 2001). To date, the Lapita settlement of site SAC is the earliest evidence of human occupation on the island of Watom, although Lapita populations had been settled on other islands in the Bismarck Archipelago since ~3400 BP (Summerhayes 2001). Newly produced dates of burials B1/WAT001 and B12/WAT003 represent a later phase of occupation, and burial B10/WAT002 yields and intermediate date of 2100 BP, showing continued use of the burial site. Additionally, a number of radiocarbon dates from floral and faunal remains, in addition to the burials, have been compiled (Anson, Green et al. 2005). An analysis of the pottery designs from the Lapita layers on Watom further supports the occupation during the Middle and Late Lapita phases in Near Oceania (Anson 1999). Petrous bone was sampled from five individuals, all of which yielded ancient DNA.

Cranial modification of individual B15/WAT006

Burial 15 was discovered in 2009, and in the osteological assessment identified as an adult male, an assessment confirmed by genetic sexing. Dating to between 2690 and 2110, but likely from the earlier period (WK-28510, Supplementary Data Table 1, S2), the person was interred during the Middle to Late Lapita period (2700 – 2200 BP).

Buried in a seated position in a round pit, archaeological and anthropological assessment of the individual provide clues for a non-Lapita related ancestry. The individual exhibits a mediolaterally narrowed and antero-posteriorly elongated form of cranial modification. Computed Tomography (CT) of the skull shows thinning of bone in the coronal suture, and the parietal is distorted inwards (towards the endocranium) (Supplementary Figure 2). Both features suggest a modification of the ‘C’, or ‘Circumferential’ type (Antón and Weinstein 1999) most likely indicating that the cranium was bound during infancy. This type of head binding has been described for individuals from Australia (Brown 1989), the Aware region of Southwest New Britain (Parkinson 1907, Blackwood and Danby 1955) and Malekula in Vanuatu (Speiser 1923).
The recovered number of genomic markers for this individual was too low to identify the source population or present-day proxies in detail, but were sufficient to show this individual had Papuan-related ancestry.

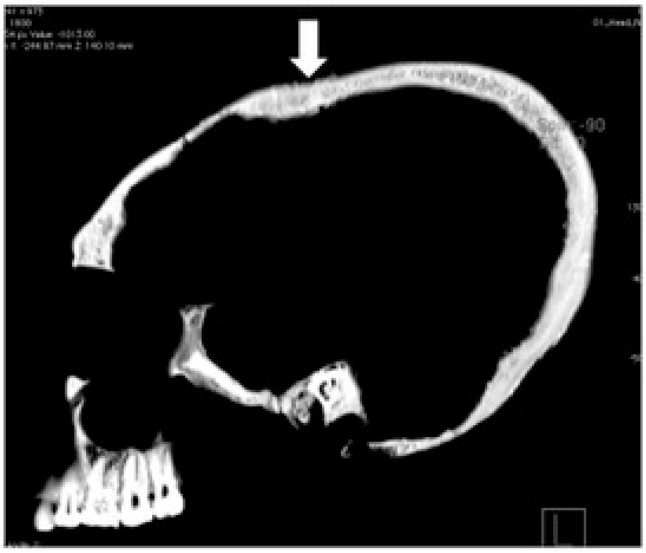

Supplementary Figure 2: Computed Tomography of the skull of the individual in Burial 15. Arrow highlights the distorted parietal.

Tilu(JCA)

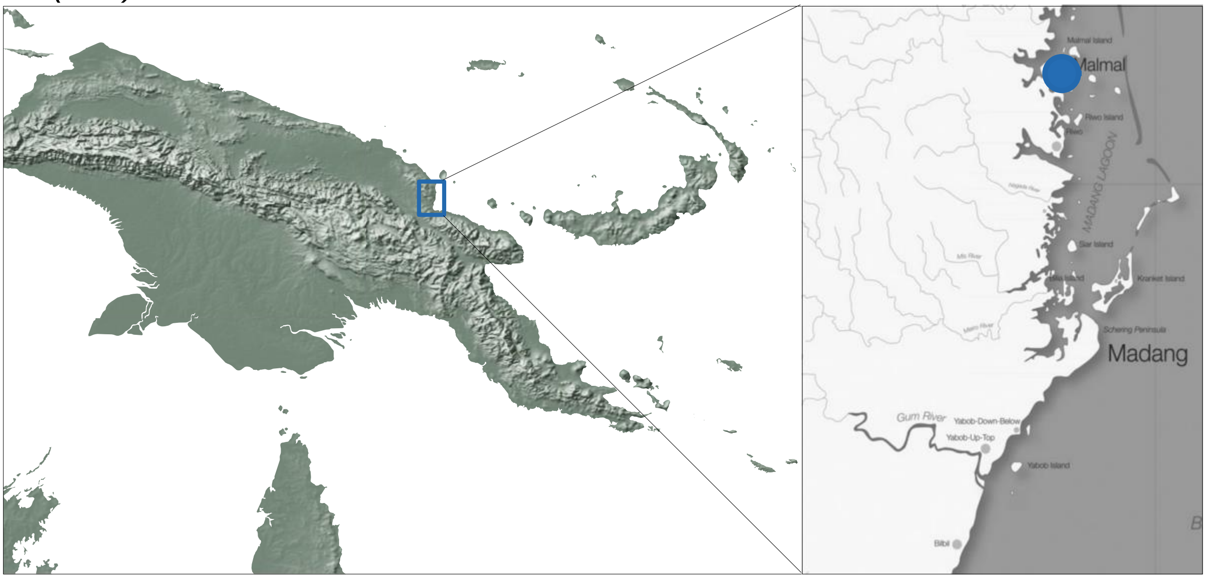

Supplementary Figure 3: Map of PNG and Coast of Madang showing location of the site.

Malmal village is situated 12 km north of Madang Township. Tilu (JCA) clan area (Egloff 1975), consists of two elongate mounds about 20 m long. Given around 3 m higher sea levels in the late Holocene (Tudhope, Buddemeier et al. 2000), Tilu may have been an island during occupation, starting about 650 BP (Egloff 1975). Seven wood charcoal samples were dated and suggest a single phase of intensive site occupation about 550–650 cal BP, consistent with other published dates for the site, but somewhat younger than the radiocarbon date retrieved from one individual produced for this study (Supplementary Data Table 2, Table 1). A single radiocarbon result from midway down the sequence dates to about 800–900 cal BP, which may reflect traces of earlier settlement or may be the result of inbuilt age (an ‘old wood’ effect) on the charcoal specimen.

Artifacts at the site comprise pottery, obsidian, animal bones from pigs and dogs, and shell artifacts. The pottery sherds show the typical styles for the Madang region. They are red-slipped, produced using paddle and anvil with hand moulding and decorated by appliqué, incision, paddle impression, and impression (Gaffney 2017). Additionally, 10 sherds from a different, unidentified, ceramic tradition were excavated.

Human remains, mostly mandibular fragments and teeth, were recovered, of which two have been analyzed in this study. Individual T1702 (TIL001) shows signs of staining indicative for betel nut (*Areca catechu*) chewing. A total of four samples were destructively sampled, of which two yielded ancient DNA.

Nunguri

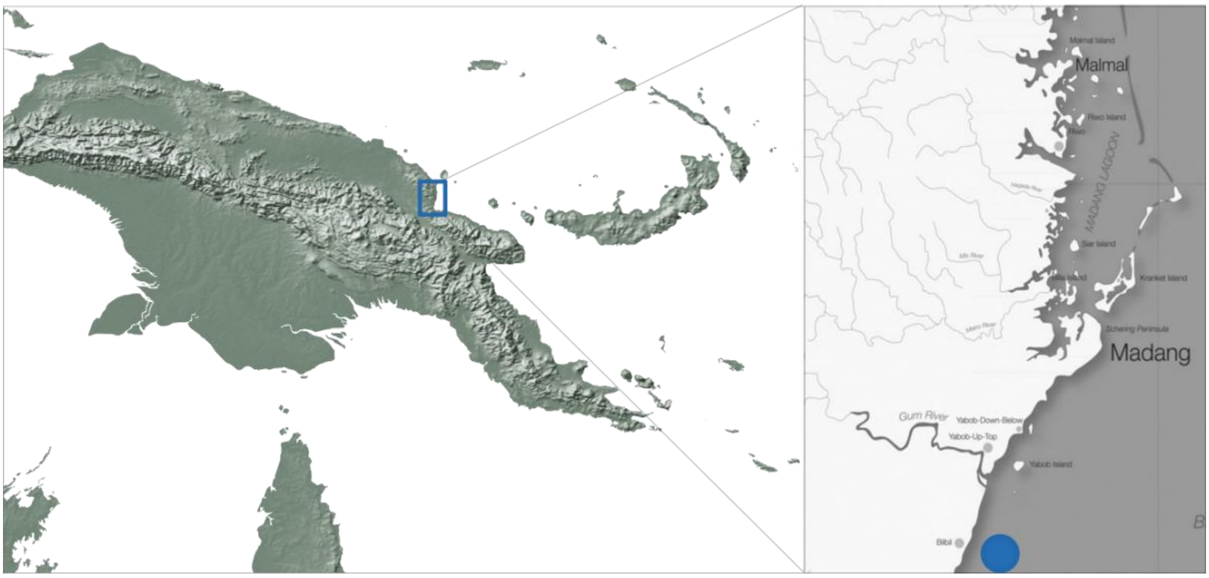

Supplementary Figure 4: Map of PNG and Coast of Madang showing location of the site.

The Nunguri site is situated in the Nunguri clan area on Bilbil Island, approximately 2 km south east off the coast of Bilbil in the Madang province. The excavation was situated on an anthropogenic mound on the western interior of the island. Six charcoal samples from the site were dated to the past 600 years (calibrated date range) (Gaffney 2020). The dominant artifact class at Nunguri was pottery sherds, but also included midden shell, lithic flakes, animal bone, tools for pottery making and pigments, shell beads and carved shell armbands. Fire cracked rocks, likely used as oven stones for cooking, showed the domestic character of the site. Human remains were sparse and restricted to spits 1 and spits 9-11 (Gaffney, Summerhayes et al. 2018).

Nebira 2 (ACJ)

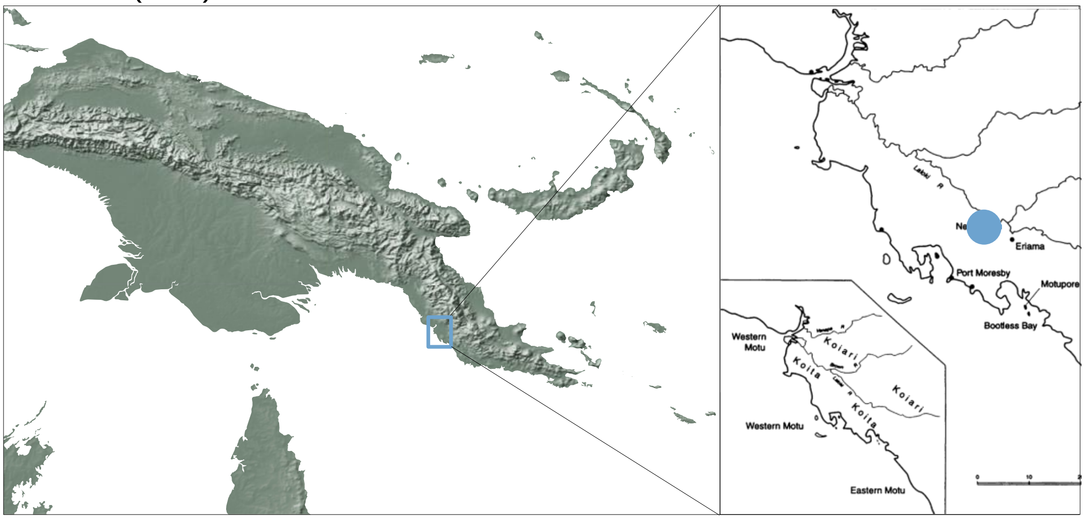

Supplementary Figure 5: Map of PNG and the South Coast showing location of sites.

Nebira is doubled peaked hill located 20 kilometers from the ocean on the South Coast of Papua New Guinea. The first excavations at in the central saddle of the Nebira peaks (site ACJ) were conducted by Dr. Susan Bulmer as rescue excavations prompted by the impending destruction of the site by quarrying (Bulmer 1978). The burials analyzed in this study were excavated from a burial ground with the site code ACJ with radiocarbon dates (Bulmer 1978) suggesting occupation between 950 – 0 cal BP. More recent radiocarbon dates (Kinaston 2010) and radiocarbon dates produced for this study (Table 1) range from 540 and 0 BP, indicating the burials were interred during latter part of the Middle Period of South Coast prehistory (A.D.1000 - ~A.D.1500) into the Protohistoric Period (~A.D.1500[1]- A.D.1875), when it was abandoned. An earlier phase of occupation of Nebira was located at the base of the hill (site Nebira 4/ACL) and there appeared to be a shift from marine subsistence (Allen 1972), to more terrestrial resources during the later occupation at the top of the hill (i.e., people interred in site ACJ). A total of 38 individuals were recovered from the ACJ site, including five individuals with non-local strontium isotope signatures (Extended Data Figure 1a,b,c) (Shaw, Buckley et al. 2011, Kinaston, Buckley et al. 2013), with varying burial practices and grave goods (Extended Data Figure 1b). Based on the stratigraphy, the burials were divided into earlier and later burial phases (Extended Data Figure 1a). Besides single burials, graves were reused for further interments and, due to the disarticulation of bones, it has been proposed that the graves had been left open for a period of time, a practice known from historical documents of the local Koita people (Allen 1977). Additionally, some bones were likely intentionally removed from the graves, a practice also documented for both groups inhabiting the region today, Motu and Koita (Allen 1977, Bulmer 1978). The pottery traditions at Nebira suggested that the earliest settlers arrived around 2000 BP and were descendants of the Lapita Cultural Complex (Allen 1977, Summerhayes and Allen 2007).

Petrous samples from a total of 26 individuals were destructively sampled, all of which yielded ancient DNA.

Eriama (ACV)

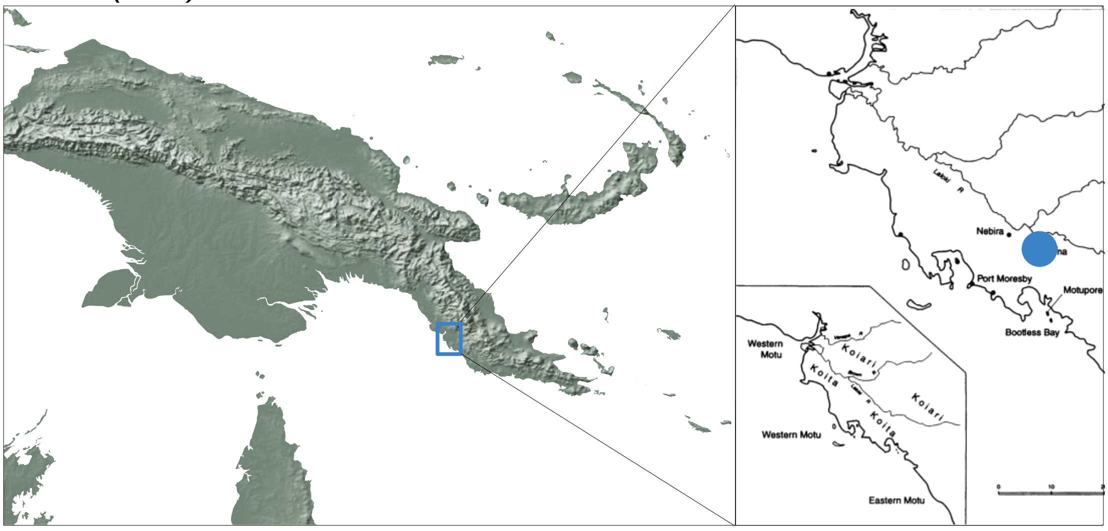

Supplementary Figure 6: Map of PNG and Coast of Madang showing location of sites.

Eriama ridge is located southeast of Nebira approximately 5 km up the Laloki river and approximately 7.2 km from the nearest point on the coast. From the 24 identified sites at Eriama, individuals analyzed in this study are from the ACV site (Bulmer 1978). Around 48-50 commingled individuals were excavated from the ACV burial cave. Both adults and non-adults were found at the site, together with shell and animal bone remains, and stone artefacts including a small piece of obsidian, possibly from Fergusson Island (Bulmer 1978). Additionally, over 1500 pottery sherds were excavated. The style of pottery associated with one burial was identified as the Waigani Style, which is a late style of bowl decoration found on Motupore Island. One cranium had evidence of painting in the form of large oval red spots. Due to the commingled nature of the burial cave, association of grave goods with individuals was impossible. It was suggested that the ACV site was only used as a place for interring secondary burials as very few hand and foot bones were found and none of the skeletons were articulated (Bulmer 1978). Inferred from the stratigraphy, the site was first inhabited from around 2000-1000 BP, and the use as burial ground commenced after that. Radiocarbon dates from charcoal and human bone indicated a use of the site beginning from 1930 ± 230 BP (GaK-2670) (Bulmer 1975). However, the Gakashuin (GaK) lab dates are now thought to be suspect. Direct radiocarbon dates from petrous bones produced for this study provided dates between 150 and 470 cal BP (MAMS 45448, MAMS 45449, MAMS 45450) (Table 1). These dates indicate that the ACV site was used for burial purposes at a contemporaneous time period as Nebira site ACJ, during the latter part of the Middle Period (A.D. 1000 - ~A.D. 1500), into the Protohistoric Period (~A.D. 1500- A.D. 1875). Unfortunately, all of the human remains from the site were mixed during the post-excavation processing of the skeletal material in Papua New Guinea. All destructively sampled individuals yielded ancient DNA, but one individual was excluded based on a recent radiocarbon date.

Material and Methods

Modelling of the split-time between Eriama and Nebira

Model Specification

To estimate genetic split times from shared IBD segments, we use the framework presented in (111). Given a demographic model, it computes the expected rate of shared IBD segments of length l by integrating over time the product of single-locus coalescent rate (denoted by $\phi(t)$) and the average number of genomic blocks of length l (denoted by $E[K_l^t]$). As in (111), we assume a simple two-island split model with no subsequent gene flow (see Supplementary Figure 2a). For this model, the single-locus coalescent rate is:

$$\phi(t) = \{0, t < T_0 \ \frac{1}{2N_0} e^{-\frac{t-T_0}{2N_0}}, t \geq T_0 \qquad (1)$$

where T_0 denotes the split time and N_0 is the (diploid) effective population size of the common ancestral population.

The model described in (111) assumes that the genetic data from both islands are collected at the same generation. However, this is not necessarily true for ancient DNA. To account for this time difference, here we modify $E[K_l^t]$ to model the situation where the genomes from two islands differ by a time difference of δt generations (we require that $\delta t < T_0$):

$$E[K_l^t; \delta t] = 2 \cdot (2t - \delta t) \cdot exp(-l(2t - \delta t)) + (G - l) \cdot (2t - \delta t)^2 \cdot exp(-l(2t - \delta t)). (2)$$

where G is the map length of a chromosome.

Integrating the product of $\phi(t)$ and $E[K_l^t; \delta t]$over t gives:

$$E[K_l] = -\frac{e^{l(\delta_t - 2T_0)}}{(4lN_0 + 1)^3}(-(G - l)(\delta_t + 4\delta_t lN_0)^2 + 2\delta_t(4lN_0 + 1)(2T_0(G - l)(4lN_0 + 1) + 4GN_0 + 1)(G - l)(4lN_0T_0 + T_0)^2 - 4T_0(4GN_0 + 1)(4lN_0 + 1) - 8N_0(4GN_0 + 1))$$

(3)

We note that for $\delta t = 0$, Eq.3 reduces to Eq.~4 in (111). The remainder of the inference of split times is as described in (111).

Modelling IBD Detection Errors

Detecting IBD segments is not perfect. To account for errors of IBD segments in empirical aDNA data, we use the same error model for IBD detection as applied in (113) and (112). Briefly, the error model considers three sources of IBD detection errors: false positive, power, and length bias. Let $\lambda(y)$ and $\hat{\lambda}(y)$ denote the true and empirically inferred rate of IBD sharing of length y, respectively. Then:

$$\hat{\lambda}(y) = FP(y) + \int_0^\infty \lambda(z)Power(z)R(y|z)dz$$

where $FP(y)$ is the false positive rate of IBD block of length y, $Power(y)$ is the power to detect a true segment of length z and $R(y|z)$ is the probability that a segment of true length z is detected being length y.

We numerically estimated the parameters of the error model from simulations mimicking typical aDNA data described in (84).

Inferring split times of Eriama and Nebira Data
After filtering out low-coverage samples, we used six samples from Nebira (NBR003, NBR004, NBR016, NBR018, NBR019, NBR025) and five samples from Eriama (ERI002, ERI006, ERI007, ERI008, ERI009) for inference. The average radiocarbon date for the six Nebira samples is 396 calBP and that of the five Eriama samples is 275 calBP. Therefore, we set $\delta t = 4$, assuming a generation time of ~28 years.

The inferred split time T_0, ancestral population size N_0, and the fitting between the observed IBD sharing rate and that predicted using the inferred model are visualized in Supplementary Figure 2b,c,d. The point estimates of T_0, N_0 are consistent when using different IBD length cutoffs, although the wide 95% confidence interval renders the point estimate largely uninformative for longer length cutoffs (e.g., $l \geq 10cM$).

Overall, our results show that the observed IBD sharing between Eriama and Nebira is described well with a two-island split model with split-time $T_0 \approx 13$ and effective population size $eN_0 \approx 550 - 580$ (or $2N_0 \approx 1100 - 1160$). However, we note that the inferred two-island split model does not necessarily reflect the true demographic history of Eriama and Nebira. Rather, the two-island split model provides a simple, parsimonious explanation of the IBD sharing data and potentially more complex models fit the inferred IBD segment sharing as well.

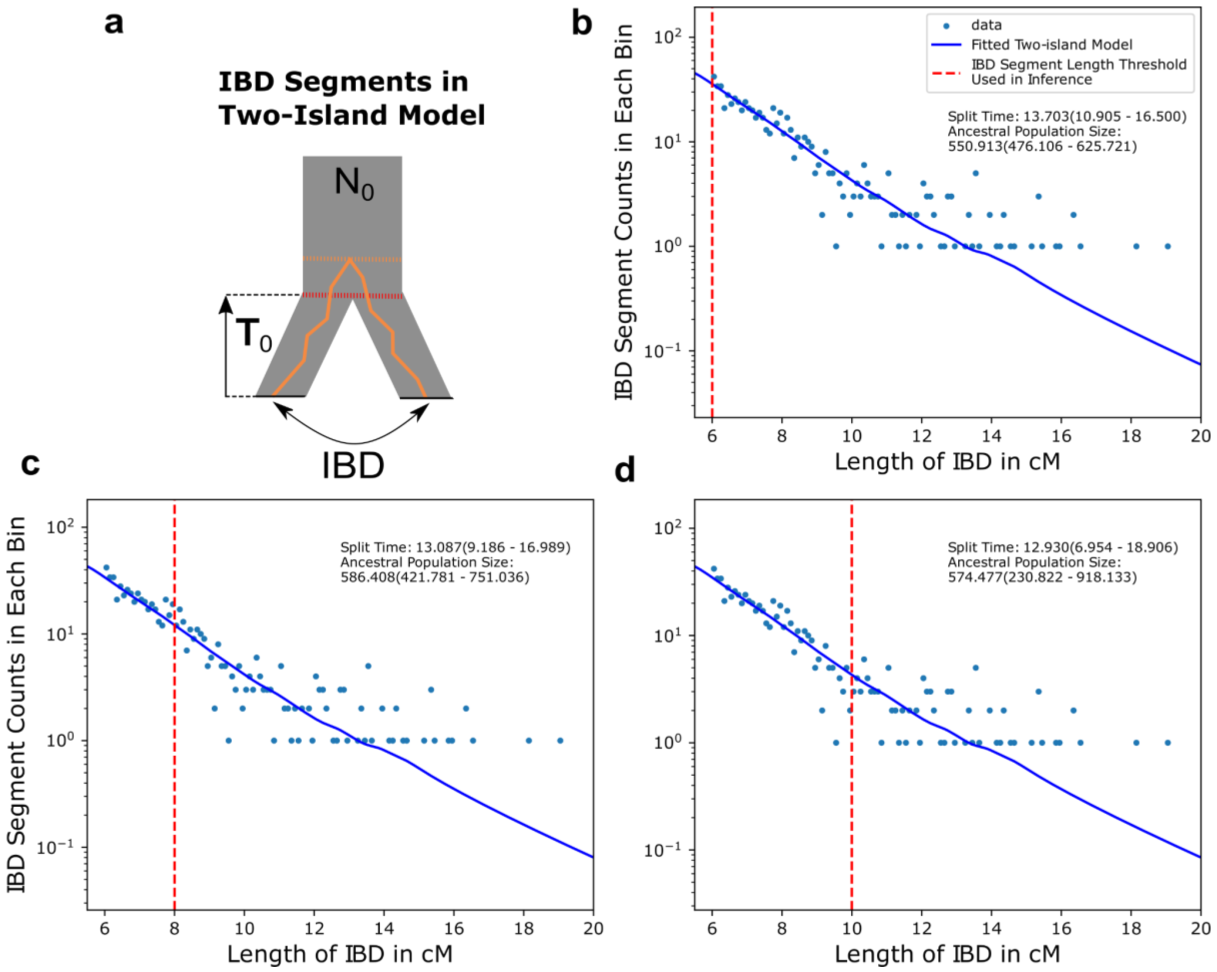

Supplementary Figure 7: **Two-island split model and its application to Eriama and Nebira Data.** (**a**): Two-island split model (without gene flow after split) and its two parameters(T_0) and (N_0). We fit the two parameters based on pairwise shared IBD segments. (**b-d**): We visualize the inferred IBD segments between Nebira and Eirama genomes, binned into different length bins (x-axis). Note the log scale on the y-axis, long IBD segments become substantially rarer. We visualize the IBD sharing predicted using the best-fit model (solid lines, error model included) for various minimum length cutoffs (vertical red lines).

References

Allen, J. (1972). "Nebira 4: an early Austronesian site in central Papua." Archaeology & Physical Anthropology in Oceania **7**(2): 92-124.
Allen, J. (1977). "Management of resources in prehistoric coastal Papua." The Melanesian Environment: 35-44.
Allen, J. (1984). "In search of the Lapita Homeland: Reconsructing the prehistory of the Bismarck Archipelag." The Journal of Pacific History **19**(4): 186-201.
Alpaslan-Roodenberg, S., D. Anthony, H. Babiker, E. Bánffy, T. Booth, P. Capone, A. Deshpande-Mukherjee, S. Eisenmann, L. Fehren-Schmitz and M. Frachetti (2021). "Ethics of DNA research on human remains: Five globally applicable guidelines." Nature **599**(7883): 41-46.
Anagnostou, P., M. Capocasa, N. Milia, E. Sanna, C. Battaggia, D. Luzi and G. Destro Bisol (2015). "When data sharing gets close to 100%: what human paleogenetics can teach the open science movement." PloS one **10**(3): e0121409.
Anson, D. (1999). "Compositional analyses of dentate-stamped Lapita and nail-incised and applied relief pottery from Watom Island." The Pacific from **5000**: 85-103.
Anson, D. (2000). "Reber-Rakival dentate-stamped motifs: documentation and comparative implications." New Zealand Journal of Archaeology **20**(1998): 119-135.
Anson, D., R. C. Green and R. K. Walter (2005). A revised and redated event phase sequence for the Reber-Rakival Lapita site, Watom Island, East New Britain Province, Papua New Guinea, Department of Anthropology, University of Otago.
Antón, S. C. and K. J. Weinstein (1999). "Artificial cranial deformation and fossil Australians revisited." Journal of Human Evolution **36**(2): 195-209.
Athens, J. S. and J. V. Ward (2004). "Holocene vegetation, savanna origins and human settlement of Guam." Records-Australian Museum: 15-30.
Athfield, N. B., R. C. Green, J. Craig, B. McFadgen and S. Bickler (2008). "Influence of marine sources on 14C ages: isotopic data from Watom Island, Papua New Guinea inhumations and pig teeth in light of new dietary standards." Journal of the Royal Society of New Zealand **38**(1): 1-23.
Bellwood, P. (2007). Southeast China and the prehistory of the Austronesians. Lost maritime cultures: China and the Pacific, Bishop Museum Press.
Bergström, A. (2023). "Improving data archiving practices in ancient genomics." bioRxiv: 2023.2005. 2015.540553.
Blackwood, B. and P. Danby (1955). "A study of artificial cranial deformation in New Britain." The Journal of the Royal Anthropological Institute of Great Britain and Ireland **85**(1/2): 173-191.
Brown, P. (1989). "Coobool Creek. A morphological and metrical analysis of the crania, mandibles and dentitions of a prehistoric Australian human population. Terra Australis, 13." Canberra: Department of Prehistory, Australian National University.
Bulmer, S. (1975). "Settlement and economy in prehistoric Papua New Guinea: a review of the archeological evidence." Journal de la Société des Océanistes **31**(46): 7-75.
Bulmer, S. (1978). Prehistoric culture change in the Port Moresby region, University of Papua New Guinea.
Carson, M. T. (2020). "Peopling of Oceania: Clarifying an initial settlement horizon in the Mariana Islands at 1500 BC." Radiocarbon **62**(6): 1733-1754.
Carson, M. T. and H. Kurashina (2012). "Re-envisioning long-distance Oceanic migration: Early dates in the Mariana Islands." World Archaeology **44**(3): 409-435.

Egloff, B. (1975). Archaeological investigations in the coastal Madang area and on Eloaue Island of the St. Matthias Group, Trustees of the Papua New Guinea Public Museum and Art Gallery.
Forsyth, M., W. Kipong, J. Barak, E. Malala, E. Kopel and I. Losoncz (2024). "Putting Data Around Intergroup Violence and Sorcery Accusation–Related Violence in Papua New Guinea."
Gaffney, D. (2017). Materialising ancestral Madang: Aspects of pre-colonial production and exchange on the northeast coast of New Guinea, University of Otago.
Gaffney, D. (2020). Materialising ancestral Madang: Pottery production and subsistence trading on the northeast coast of New Guinea, University of Otago Studies in Archaeology· No. 29.
Gaffney, D., G. R. Summerhayes, M. Mennis, T. Beni, A. Cook, J. Field, G. Jacobsen, F. Allen, H. Buckley and H. Mandui (2018). "Archaeological investigations into the origins of Bel trading groups around the Madang coast, northeast New Guinea." The Journal of Island and Coastal Archaeology **13**(4): 501-530.
Green, R. (1991). "Near and Remote Oceania: disestablishing" Melanesia" in culture history." Man and a half: Essays in Pacific Anthropology and Ethnobotany in honour of Ralph Bulmer.
Green, R. and D. Anson (2000). "Archaeological investigations on Watom Island: early work, outcomes of recent investigations and future prospects." New Zealand Journal of Archaeology **20**(1998): 183-197.
Green, R., D. Anson and J. Specht (1989). "The SAC burial ground, Watom Island, Papua New Guinea." Records of the Australian Museum **41**(3): 215-221.
Hung, H.-c., M. T. Carson, P. Bellwood, F. Z. Campos, P. J. Piper, E. Dizon, M. J. L. A. Bolunia, M. Oxenham and Z. Chi (2011). "The first settlement of Remote Oceania: the Philippines to the Marianas." Antiquity **85**(329): 909-926.
Kabutaulaka, T. (2015). "Re-Presenting Melanesia: Ignoble Savages and Melanesian Alter-Natives." EDITED BY STEWART FIRTH: 193.
Kinaston, R. (2010). Prehistoric diet and health in the western Pacific Islands, University of Otago.
Kinaston, R., H. Buckley, A. Gray, B. Shaw and H. Mandui (2013). "Exploring subsistence and cultural complexes on the south coast of Papua New Guinea using palaeodietary analyses." Journal of Archaeological Science **40**(2): 904-913.
Lipson, M., P. Skoglund, M. Spriggs, F. Valentin, S. Bedford, R. Shing, H. Buckley, I. Phillip, G. K. Ward and S. Mallick (2018). "Population turnover in Remote Oceania shortly after initial settlement." Current Biology **28**(7): 1157-1165. e1157.
Liu, Y.-C., R. Hunter-Anderson, O. Cheronet, J. Eakin, F. Camacho, M. Pietrusewsky, N. Rohland, A. Ioannidis, J. S. Athens and M. T. Douglas (2022). "Ancient DNA reveals five streams of migration into Micronesia and matrilocality in early Pacific seafarers." Science **377**(6601): 72-79.
Parkinson, R. (1907). Thirty Years in the South Seas: land and people, customs and traditions in the Bismarck Archipelago and on the German Solomon Islands (trans. by Dennison, John & J. Peter White). Bathurst, NSW: Crawford House Publishing.(Dreißig Jahre in der Südsee: Land und Leute ….
Petchey, F. and G. Clark (2021). "Clarifying the age of initial settlement horizon in the Mariana Islands and the impact of hard water: A response to Carson (2020)." Radiocarbon **63**(3): 905-913.
Petchey, F., G. Clark, I. Lindeman, P. O'Day, J. Southon, K. Dabell and O. Winter (2018). "Forgotten news: Shellfish isotopic insight into changing sea-level and

associated impact on the first settlers of the Mariana Archipelago." Quaternary Geochronology **48**: 180-194.
Petchey, F. and R. Green (2005). "Use of three isotopes to calibrate human bone radiocarbon determinations from Kainapirina (SAC), Watom Island, Papua New Guinea." Radiocarbon **47**(2): 181-192.
Petchey, F., M. Spriggs, F. Leach, M. Seed, C. Sand, M. Pietrusewsky and K. Anderson (2011). "Testing the human factor: radiocarbon dating the first peoples of the South Pacific." Journal of Archaeological Science **38**(1): 29-44.
Pugach, I., A. Hubner, H.-C. Hung, M. Meyer, M. T. Carson and M. Stoneking (2021). "Ancient DNA from Guam and the Peopling of the Pacific." Proceedings of the National Academy of Sciences **118**(1).
Shaw, B., H. Buckley, G. Summerhayes, C. Stirling and M. Reid (2011). "Prehistoric migration at Nebira, South Coast of Papua New Guinea: New insights into interaction using isotope and trace element concentration analyses." Journal of Anthropological Archaeology **30**(3): 344-358.
Skoglund, P., C. Posth, K. Sirak, M. Spriggs, F. Valentin, S. Bedford, G. R. Clark, C. Reepmeyer, F. Petchey and D. Fernandes (2016). "Genomic insights into the peopling of the Southwest Pacific." Nature **538**(7626): 510-513.
Speiser, F. (1923). "Ethnology of Vanuatu." An Early Twentieth Century Study.
Summerhayes, G. R. (2001). "Defining the chronology of Lapita in the Bismarck Archipelago." The chronology of Lapita dispersal in Oceania.
Summerhayes, G. R. and J. Allen (2007). "Lapita writ small? Revisiting the Austronesian colonisation of the Papuan south coast." Oceanic Explorations.
Terrell, J. (1986). "Causal pathways and causal processes: Studying the evolutionary prehistory of human diversity in language, customs, and biology." Journal of Anthropological Archaeology **5**(2): 187-198.
Thomas, N., A. Abramson, I. Brady, R. Green, M. Sahlins, R. A. Stephenson, F. Valjavec and R. G. White (1989). "The force of ethnology: Origins and significance of the Melanesia/Polynesia division [and comments and replies]." Current Anthropology **30**(1): 27-41.
Tudhope, A. W., R. W. Buddemeier, C. P. Chilcott, K. R. Berryman, D. G. Fautin, M. Jebb, J. H. Lipps, R. G. Pearce, T. P. Scoffm and G. B. Shimmield (2000). "Alternating seismic uplift and subsidence in the late Holocene at Madang, Papua New Guinea: evidence from raised reefs." Journal of Geophysical Research: Solid Earth **105**(B6): 13797-13807.

Supplementary Tables

Available as separate data sheets

Supplementary Table 1**:** Summary of the individuals analysed in this study. Details on site, location, Archaeological and lab IDs, C14-dating, Sr-values published in Shaw et al 2015, Libraries, genetic sex, contamination estimates, uniparental haplogroups and coverage on the 1240K SNP panel. Site locations are approximations, not the exact location of the site

Supplementary Table 2**:** Newly produced radiocarbon Dates (produced in the Kurt-Engelhorn Zentrum für Archäometrie, Mannheim) and Isotopic values for the individuals included in this study. Detailed are the site, Lab IDs from MPI-EVA, Dating Labs, Archaeological ID, Element sampled, unclaibraed C14 age (CRA (BP)) and standard error (p/m), 68% probability and 95% probability, range from – to for 95% probability range; Stable Isotope Lab ID, %N, %C, C:N, $\delta^{15}N$ (‰), $\delta^{13}C$ (‰).

Supplementary Table 3**:** Comparisons of the stable Isotope analysis for different elements of individuals from Nebira. Detailed are Dating ID/Previously published ID, MPI ID, Archaeological ID, Element sampled, δ15N (‰), %N, δ13C (‰), %C , C:N ratio and use.

Supplementary Table 4: Summary of the newly produced and published stable isotope data considered for this study. Detailed are Site ID, Burial number/Archaeological ID, Element sampled, Lab ID, Period (as described in (34)), Age at death, Sex determination (Karyotype as inferred from DNA analysis, F, M, UK (unknown) or n/a (for subadults)), Bone $\delta^{15}N$ (‰) D, %N, Bone $\delta^{13}C$ (‰) D, %C, C:N, δ34S (‰), %S, N:S, C:S, Dentine $\delta^{15}N$ (‰)D, %N, Dentine $\delta^{13}C$ (‰), %C, C:N, Enamel carb $\delta^{13}C$ (‰) E, 87Sr/86SrF

Supplementary Table 5: Stable isotope values of Wallabys samples at Eriama and Nebira as a comparatibve measure for the local signal at the sites. Detailed are the site, Lab ID, context number, element sampled, $\delta^{15}N$ dentine (‰), %N, $\delta^{13}C$ dentine (‰), %C , C:N ratio, δ13C $(‰)_{enamel}$, SR, SE

Supplementary Table 6: Ward's hierarchical cluster analysis of dietary isotope results for humans (a) and animals (b).

Supplementary Table 7: Microparticle content of human dental calculus samples from Watom.

Supplementary Table 8**:** Sequencing summaries for initial screening (SG), 1240K capture (TF) and mitochondrial capture (mt), detailing Sequencing depth, endogenous

DNA content on average and on target, coverage statistics, mitochondrial to nuclear ratio, damage observed, fragment lengths and GC content.

Supplementary Table 9: Sex determination and contamination estimation through ANGSD for nuclear contamination in males, and through schmutzi assessing the contamination based on mitochondrial data.

Supplementary Table 10: principal component analysis, present-day populations used to calculate the principal components, results for the principal components 1-10.

Supplementary Table 11: f-statistics investigating differential affinities between Early Remote Oceanians (ERO) and ancient Austronesians (Suogang); New Guinea and populations from the Bismarck Archipelago, and details on the Papuan ancestry in Tilu.

Supplementary Table 12**:** ancestry modeling with qpWave/qpAdm. Details on the test scores of individuals against their respective group, the model of Asian (Ami) and Near Oceanic (New_Guinea) ancestry and testing continuity with ancient individuals from Watom and the present-day inhabitants (Tilu). "ID" denotes the respective individual tested regarding its ancestry consistent with "source", where the fit of this model is given in the "p-value" (<0.05 considered a fitting model, accepting the ancestry of "ID" is not distinguishable from "source" in respect to the "Reference community"). Individuals where p-value is > 0.05, 1 wave is rejected and the "ID" further modeled with qpAdm, modeling with "%Ami" and "%New_Guinea". A p-value >0.05 indicates a fitting model, test are one-sided, and no adjustments are made for multiple comparisons as is standard in the field; SE computed through block jackknive.

Supplementary Table 13: Admixture Dating. Dating of admixture between the Asian related and Papuan-related ancestry components. Papuan component modeled through Papuan.SG, Asian component through a combination of Han.DG, Ami.DG, Atayal.DG, Igorot.DG, Kinh.DG, Dai.DG, She.DG

Supplementary Table 14**:** Genetic relatedness analysis. A. degree of genetic relatedness calculated with READ. Determination of first (parent-offspring; siblings) and second degree (Aunt/Uncle - Nephew/Niece; Grandparent - Grandchildren). B. Identity-by-descent analysis. Length and number of shared IBD blocks as calculated with ancIBD, partitioned according to minimum length of IBD blocks in cM.
