# Supplementary material for: The impact of human dispersals and local interactions on the genetic diversity of coastal Papua New Guinea over the past 2,500 years
Source: Nat Ecol Evol. 2025 Jun 4;9(6):908–23. doi: 10.1038/s41559-025-02710-x (PMC12148941; doi:10.1038/s41559-025-02710-x)
Supplement: Supplementary file 2 — Reporting Summary [file 41559_2025_2710_MOESM2_ESM.pdf]

nature portfolio

Corresponding author(s): Kathrin Nägele, Rebecca Kinaston, Cosimo Posth, Johannes Krause

Last updated by author(s): 10/31/2024

Reporting Summary

Nature Portfolio wishes to improve the reproducibility of the work that we publish. This form provides structure for consistency and transparency in reporting. For further information on Nature Portfolio policies, see our Editorial Policies and the Editorial Policy Checklist.

Statistics

For all statistical analyses, confirm that the following items are present in the figure legend, table legend, main text, or Methods section.

n/a	Confirmed	
☐	☒	The exact sample size (*n*) for each experimental group/condition, given as a discrete number and unit of measurement
☐	☒	A statement on whether measurements were taken from distinct samples or whether the same sample was measured repeatedly
☐	☒	The statistical test(s) used AND whether they are one- or two-sided<br>*Only common tests should be described solely by name; describe more complex techniques in the Methods section.*
☐	☒	A description of all covariates tested
☐	☒	A description of any assumptions or corrections, such as tests of normality and adjustment for multiple comparisons
☐	☒	A full description of the statistical parameters including central tendency (e.g. means) or other basic estimates (e.g. regression coefficient) AND variation (e.g. standard deviation) or associated estimates of uncertainty (e.g. confidence intervals)
☐	☒	For null hypothesis testing, the test statistic (e.g. *F*, *t*, *r*) with confidence intervals, effect sizes, degrees of freedom and *P* value noted<br>*Give P values as exact values whenever suitable.*
☒	☐	For Bayesian analysis, information on the choice of priors and Markov chain Monte Carlo settings
☒	☐	For hierarchical and complex designs, identification of the appropriate level for tests and full reporting of outcomes
☒	☐	Estimates of effect sizes (e.g. Cohen's *d*, Pearson's *r*), indicating how they were calculated

Our web collection on statistics for biologists contains articles on many of the points above.

Software and code

Policy information about availability of computer code

Data collection	n/a
Data analysis	for details and citations see Supplementary Information. Clip&Merge, AdapterRemoval v.2, Burrows–Wheeler Aligner (BWA) v. 0.7.12, DeDup v. 0.12.2, SAMtools v. 1.3, pileupCaller v.8.6.5, DamageProfiler v0.3.1 , ANGSD v. 0.919 , Schmutzi, ADMIXTURE v.1.3.0 , smartpca v. 13050 , qp3Pop v. 5.0 , qpDstat v.5.0 , qpWave v. 410 , qpAdm v. 5.0 , DATES, nf-core/eager v. 2.4.0 , Nextflow v. 21.04.3, FastQC v. 0.11.9, AdapterRemoval v. 2.3.2, SAMtools version 1.12, Qualimap version 2.2.2-dev , MarkDuplicates version 2.26.0 , endorS.py version 0.4, DamageProfiler version 0.4.9, Geneious version 2019.2.3, MultiQC version 1.11 , HaploGrep version 2.4.0 , hapROH v.0.3a4 , Jupyter notebooks v.6.4.4, READ, ATLAS v.0.9 , GLIMPSE v.1.0.0, ancIBD v.0.2a2 , OxCal 4.4, https://github.com/hyl317/two_island

For manuscripts utilizing custom algorithms or software that are central to the research but not yet described in published literature, software must be made available to editors and reviewers. We strongly encourage code deposition in a community repository (e.g. GitHub). See the Nature Portfolio guidelines for submitting code & software for further information.

Data

Policy information about availability of data

All manuscripts must include a data availability statement. This statement should provide the following information, where applicable:

- Accession codes, unique identifiers, or web links for publicly available datasets
- A description of any restrictions on data availability
- For clinical datasets or third party data, please ensure that the statement adheres to our policy

The raw data of the captured libraries is available at the European Nucleotide Archive (ENA) https://www.ebi.ac.uk/PRJEB68153 under the accession number PRJEB68153. The genotypes of the newly published individuals can be sourced through the Poseidon framework community archive https://github.com/poseidon-framework/community-archive under 2024_NaegeleNatureEcologyEvolution.
The skeletal elements sampled at the Max-Planck-Institute of Geoanthropology in Jena, Germany, will be returned to the National Museum and Art Gallery of Papua New Guinea in Port Moresby (Nunguri and Tilu) and to the Department of Anatomy, University of Otago, Aotearoa (Nebira, Eriama and Watom) IN SPRING of 2025 to be curated with their respective skeletal assemblages.

Research involving human participants, their data, or biological material

Policy information about studies with human participants or human data. See also policy information about sex, gender (identity/presentation), and sexual orientation and race, ethnicity and racism.

Reporting on sex and gender	karyotypes of individuals were determined to inform X-and Y-chromosome specific analysis, i.e. sex-biased admixture and Y-chromosmal contamination, haplogroups.
Reporting on race, ethnicity, or other socially relevant groupings	n/a
Population characteristics	there were no co-variate relevant population characteristics
Recruitment	selection of individuals was determined by availability of archaeological material and preservation of DNA
Ethics oversight	Papua New Guinea National Art Gallery and Museum

Note that full information on the approval of the study protocol must also be provided in the manuscript.

Field-specific reporting

Please select the one below that is the best fit for your research. If you are not sure, read the appropriate sections before making your selection.

☒ Life sciences ☐ Behavioural & social sciences ☐ Ecological, evolutionary & environmental sciences

For a reference copy of the document with all sections, see nature.com/documents/nr-reporting-summary-flat.pdf

Life sciences study design

All studies must disclose on these points even when the disclosure is negative.

Sample size	was determined by preservation of DNA and number of individuals excavated and curated from a site
Data exclusions	one individual was excluded from the analysis based on a radiocarbon date overlapping with the present
Replication	the results were replicated within our team, but not shared with an external analyst for replication
Randomization	n/a
Blinding	n/a

Reporting for specific materials, systems and methods

We require information from authors about some types of materials, experimental systems and methods used in many studies. Here, indicate whether each material, system or method listed is relevant to your study. If you are not sure if a list item applies to your research, read the appropriate section before selecting a response.

Materials & experimental systems

n/a	Involved in the study
☒	☐ Antibodies
☒	☐ Eukaryotic cell lines
☐	☒ Palaeontology and archaeology
☒	☐ Animals and other organisms
☒	☐ Clinical data
☒	☐ Dual use research of concern
☒	☐ Plants

Methods

n/a	Involved in the study
☒	☐ ChIP-seq
☒	☐ Flow cytometry
☒	☐ MRI-based neuroimaging

Palaeontology and Archaeology

Specimen provenance

oversight of archaeological material, including human remains lies with the Papua New Guinea National Museum and Art Gallery. Permission for this study was granted to Hallie Buckley and Glenn Summerhayes.

Specimen deposition

The skeletal elements sampled at the Max-Planck-Institute of Geoanthropology in Jena, Germany, will be returned to the National Museum and Art Gallery of Papua New Guinea in Port Moresby (Nunguri and Tilu) and to the Department of Anatomy, University of Otago, Aotearoa (Nebira, Eriama and Watom) at the beginning of 2024 to be curated with their respective skeletal assemblages

Dating methods

AMS dates for this study were produced at the Curt-Engelhorn-Zentrum Archäometrie gGmbH in Mannheim, Germany. Collagen from bone and dentin was extracted using a modified Longin method and long molecules removed with ultrafiltration before freeze-drying the product. After the catalytic reduction to graphite the 14C content was measured with an AMS-System type MICADAS. The isotopic ratios of 14C/12C and 13C/12C of samples, standards (Oxalic acid II) and controls were measured simultaneously. The resulting 14C dates were normed with δ13C=-25‰ and calibrated using using Intcal20, OxCal 4.4, and marine20. The radiocarbon dates and quality collagen indicators (collagen yields, C/N ratios, %C and %N) are reported in Table S2 of the supplementary information

☒ Tick this box to confirm that the raw and calibrated dates are available in the paper or in Supplementary Information.

Ethics oversight

Papua New Guinea National Museum and Art Gallery.

Note that full information on the approval of the study protocol must also be provided in the manuscript.

Plants

Seed stocks

n/a

Novel plant genotypes

n/a

Authentication

n/a
